# Antibacterial activity of varying UMF-graded Manuka honeys

**DOI:** 10.1371/journal.pone.0224495

**Published:** 2019-10-25

**Authors:** Alodia Girma, Wonjae Seo, Rosemary C. She

**Affiliations:** Department of Pathology, Keck School of Medicine of the University of Southern California, Los Angeles, California, United States of America; University of Messina, ITALY

## Abstract

Honey has been used as a traditional remedy for skin and soft tissue infections due to its ability to promote wound healing. Manuka honey is recognized for its unusually abundant content of the antibacterial compound, methylglyoxal (MGO). The Unique Manuka Factor (UMF) grading system reflects the MGO concentration in Manuka honey sold commercially. Our objective was to observe if UMF values correlated with the antibacterial activity of Manuka honey against a variety of pathogens purchased over the counter. The antibacterial effect of Manuka honey with UMF values of 5+, 10+, and 15+ from the same manufacturer was assessed by the broth microdilution method. Minimum inhibitory concentration (MIC) values were determined against 128 isolates from wound cultures representing gram-positive, gram-negative, drug-susceptible, and multi-drug resistant (MDR) organisms. Lower MICs were observed with UMF 5+ honey for staphylococci (n = 73, including 25 methicillin-resistant *S*. *aureus*) and *Pseudomonas aeruginosa* (n = 22, including 10 MDR) compared to UMF 10+ honey (p<0.05) and with UMF 10+ compared to UMF 15+ (p = 0.01). For Enterobacteriaceae (n = 33, including 14 MDR), MIC values were significantly lower for UMF 5+ or UMF 10+ compared to UMF 15+ honey (p<0.01). MIC_50_ for UMF 5+, UMF 10+, and UMF 15+ honey against staphylococci was 6%, 7%, and 15%, and for Enterobacteriaceae was 21%, 21%, and 27%, respectively. For *Pseudomonas aeruginosa* MIC_50_ was 21% and MIC_90_ was 21–27% for all UMFs. Manuka honey exhibited antimicrobial activity against a spectrum of organisms including those with multi-drug resistance, with more potent activity overall against gram-positive than gram-negative bacteria. Manuka honey with lower UMF values, in our limited sampling, paradoxically demonstrated increased antimicrobial activity among the limited samples tested, presumably due to changes in MGO content of honey over time. The UMF value by itself may not be a reliable indicator of antibacterial effect.

## Background

Honey has long been used as a wound salve and has been found experimentally to stimulate tissue regeneration, facilitate wound debridement, reduce inflammation, and exert antibacterial properties [1]. Its antibacterial effects arise from its low pH, ability to dehydrate bacteria, and phytochemical content [2]. Manuka honey, derived from flowers of the Manuka bush (*Leptospermum scoparium*), in particular has been noted for its bactericidal activity. Many types of honey contain hydrogen peroxide as the main antimicrobial mechanism, whereas the antibacterial effects of Manuka honey are considered to be primarily from its substantial content of methylglyoxal (MGO), a compound found in only certain honeys [3, 4].

MGO is a compound formed from the dehydration of dihydroxyacetone, a natural phytochemical within *Leptospermum* flower nectar [5]. It has demonstrated selective toxicity to bacterial cells when applied to wounds, and has separately been shown to cause bacterial cell lysis, inhibit flagellation, and disrupt bacterial cell division [6–8]. The concentration of MGO in Manuka honey correlates strongly with antibacterial activity [9–11]. Additional phytochemicals, such as phenolic compounds, flavonoids, and defensins likely contribute synergistically as MGO by itself does not achieve the same level of antibacterial activity as Manuka honey of equal MGO concentration [7, 12, 13]. Nonetheless, MGO is still regarded as the major antimicrobial constituent and various Manuka honey grading schemes for commercially sold honey are based in large part on MGO concentrations. One grading system, termed Unique Manuka Factor (UMF), was originally developed to express the antibacterial activity of a Manuka honey in units equivalent to % phenol against *Staphylococcus aureus* in an agar well diffusion assay [14]. With discovery of MGO and its role in antimicrobial activity in Manuka honey, UMF grade is now primarily based on the measured level of MGO such that UMF 5+ honey has ≥ 83 mg/kg MGO, UMF 10+ has ≥ 263 mg/kg MGO, and UMF 15+ has ≥ 514 mg/kg MGO [15]. Manuka honey with higher UMF are presumed to have more potent antibacterial properties and are more expensive in the consumer market [3]. Given the widespread use of MGO content as an indicator of Manuka honey grade and the wide acceptance of MGO as the primary antibiotic compound in Manuka honey, our objective was to observe if UMF values correlated with the antibacterial activity of Manuka honey purchased over the counter against a variety of clinically relevant bacterial isolates.

## Materials and methods

### Bacterial isolates

Isolates originated from wound cultures of clinical specimens performed in the clinical microbiology laboratories of Keck Medical Center of the University of Southern California and LAC+USC Medical Center (Los Angeles, CA). Both fresh subcultures and frozen isolates were included. From frozen glycerol stocks, organisms were subcultured two times before being used for antimicrobial susceptibility testing [16]. Each isolate was previously identified by matrix-assisted laser desorption ionization time-of-flight (MALDI-TOF) mass spectrometry (Vitek MS, bioMérieux, St. Louis, MO) and undergone susceptibility testing (Vitek 2, bioMérieux) according to routine clinical protocol. Carbapenemase status for Enterobacteriaceae was determined on the basis of PCR detection of carbapenemase genes (Xpert Carba-R, Cepheid, Sunnyvale, CA). A total of 128 bacterial organisms were selected for antimicrobial susceptibility testing: 48 *Staphylococcus aureus* (25 methicillin-resistant *S*. *aureus* (MRSA) and 23 methicillin-susceptible *S*. *aureus* (MSSA)), 25 coagulase-negative staphylococci (11 *S*. *epidermidis*, 5 *S*. *lugdunensis*, 5 *S*. *hominis*, 2 *S*. *capitis*, 1 *S*. *warneri*, and 1 *S*. *saccharolyticus*), 33 enteric gram-negative bacilli (17 *Klebsiella pneumoniae* including 9 *bla*_KPC_ carbapenemase producers, 1 carbapenem-resistant but carbapenemase-negative strain, and 1 extended-spectrum beta-lactamase (ESBL) producer; 11 *E*. *coli* including 3 ESBL producers; 1 *K*. *aerogenes*, and 4 *Enterobacter* sp.), and 22 *Pseudomonas aeruginosa* (10 multi-drug resistant (MDR) and 12 non-MDR). MDR status was determined using Centers for Disease Control and Prevention definitions [17].

### Antimicrobial susceptibility testing

Manuka honeys graded UMF 5+, 10+, and 15+ (Comvita New Zealand LTD) were used in this study within 6 months of purchase and prior to the expiration date. One sample of each UMF grade was used and expiration dates were all within the same 2-month period (Sept to Nov 2020). For each UMF-graded honey, we applied the broth microdilution method following the Clinical and Laboratory Standards Institute (CLSI) guidelines to assess minimal inhibitory concentrations (MIC) of antibacterial agents [16]. Stock solutions were prepared prior to each batch of testing by preparing up to 60% (w/v) honey in Mueller-Hinton broth (Remel Inc., Lenexa, KS). Solutions were vortexed until completely dissolved, then sterilized by serial filtration through 0.45 μm and 0.22 μm polyvinylidene fluoride (PVDF) membranes (MilliporeSigma, Burlington, MA) to eliminate contaminating spore-forming organisms. Based on expected MIC values from preliminary results, we tested Manuka honey concentrations (% w/v) of 5%, 6%, 7%, 8%, 9%, 10%, and 15% for gram-positive organisms and 9%, 15%, 21%, 27%, 33%, 39%, and 45% for gram-negative organisms. The colony suspension method was used for preparing organism inocula from blood agar media after 18–24 hr subculture. Dilutions of a 0.5 McFarland suspension of each organism were made to a final organism concentration of ~5 x 10^4^ colony forming units/mL in a final test volume of 0.1 mL per well on a 96-well plate. Each organism was tested against all three UMF-graded honeys in parallel using the same organism preparation. MICs were read after 20–24 h incubation at 35°C in ambient air for bactericidal activity. Growth and sterility controls were included for each organism-honey combination. Purity of each organism suspension was assessed by subculturing an aliquot onto blood agar plates. Any failed controls, tests with multiple skipped wells, or mixed purity check cultures resulted in repeat testing with a fresh subculture of the organism.

### Statistical analysis

MIC results at the 50^th^ percentile (MIC_50_) and the 90^th^ percentile (MIC_90_) were analyzed for each UMF and organism group. MIC values of the different UMF honeys tested against the same organisms underwent pairwise comparisons by the two-tailed Wilcoxon signed-rank test. MIC values for different organism groups tested by the same UMF-graded honey were compared using the Mann-Whitney U test. Results were considered statistically significant if p<0.05 (GraphPad Prism v8).

## Results

Gram-negative organisms demonstrated distributions of MIC values that were significantly higher than for staphylococci (p<0.01 for each UMF grade of honey). MIC_50_ values for Gram-negative organisms were ≥ 21% compared to 5–15% for staphylococci and the different UMF honeys. Summary statistics organism groups are shown in Tables 1 and 2 and individual organism results can be found in S1 Table.

**Table 1:** MIC_50_, MIC_90_, and MIC ranges of Manuka honeys UMF 5+, 10+, and 15+ tested against MRSA, MSSA, and coagulase-negative staphylococci.

Organism		UMF 5+	UMF 10+	UMF 15+
MRSA (n = 25)	MIC_50_ (% w/v)	6	7	15
	MIC_90_ (% w/v)	8	8	15
	MIC range (% w/v)	≤5 to >15	≤5 to >15	7 to >15
MSSA (n = 23)	MIC50 (% w/v)	6	7	15
	MIC90 (% w/v)	7	8	15
	MIC range (% w/v)	≤5 to 7	≤5 to 10	9 to >15
Coagulase-negative staphylococci (n = 25)	MIC50 (% w/v)	6	7	10
	MIC90 (% w/v)	7	8	15
	MIC range (% w/v)	≤5–8	≤5–10	6–15
All *Staphylococcus* spp. (n = 73)	MIC50 (% w/v)	5	6	15
	MIC90 (% w/v)	7	8	15
	MIC range (% w/v)	≤5 to >15	≤5 to >15	6 to >15

MIC, minimal inhibitory concentration; MRSA, methicillin-resistant *Staphylococcus aureus*; MSSA, methicillin-susceptible *S*. *aureus*.

**Table 2:** MIC_50_, MIC_90_, and MIC ranges of Manuka honeys UMF 5+, 10+, and 15+ tested against gram-negative organisms.

Organisms			UMF 5+	UMF 10+	UMF 15+
*Pseudomonas aeruginosa*	All (n = 22)	MIC_50_ (% w/v)	21	21	21
		MIC_90_ (% w/v)	21	27	27
		MIC range (% w/v)	≤9–27	15–27	21–33
	MDR (n = 10)	MIC_50_ (% w/v)	15	21	21
		MIC_90_ (% w/v)	21	21	21
		MIC range (% w/v)	≤9–21	15–27	21–27
	Non-MDR (n = 12)	MIC_50_ (% w/v)	21	21	27
		MIC_90_ (% w/v)	21	27	33
		MIC range (% w/v)	15–27	15–27	21–33
Enterobacteriaceae	All (n = 33)	MIC_50_ (% w/v)	21	21	27
		MIC_90_ (% w/v)	33	33	33
		MIC range (% w/v)	15–33	15–33	21–33
	ESBL, CRE (n = 14)	MIC_50_ (% w/v)	27	33	27
		MIC_90_ (% w/v)	33	33	33
		MIC range (% w/v)	21–33	21–33	21–33
	Non-ESBL/CRE (n = 19)	MIC_50_ (% w/v)	21	21	27
		MIC_90_ (% w/v)	27	27	33
		MIC range (% w/v)	15–27	15–27	21–39
All Gram-negative organisms (n = 55)		MIC_50_ (% w/v)	21	21	27
		MIC_90_ (% w/v)	33	33	33
		MIC range (% w/v)	9–33	15–33	21–39

MIC, minimal inhibitory concentration; ESBL, extended-spectrum beta-lactamase; CRE, carbapenem-resistant Enterobacteriaceae; MDR, multi-drug resistant.

**Fig 1:**
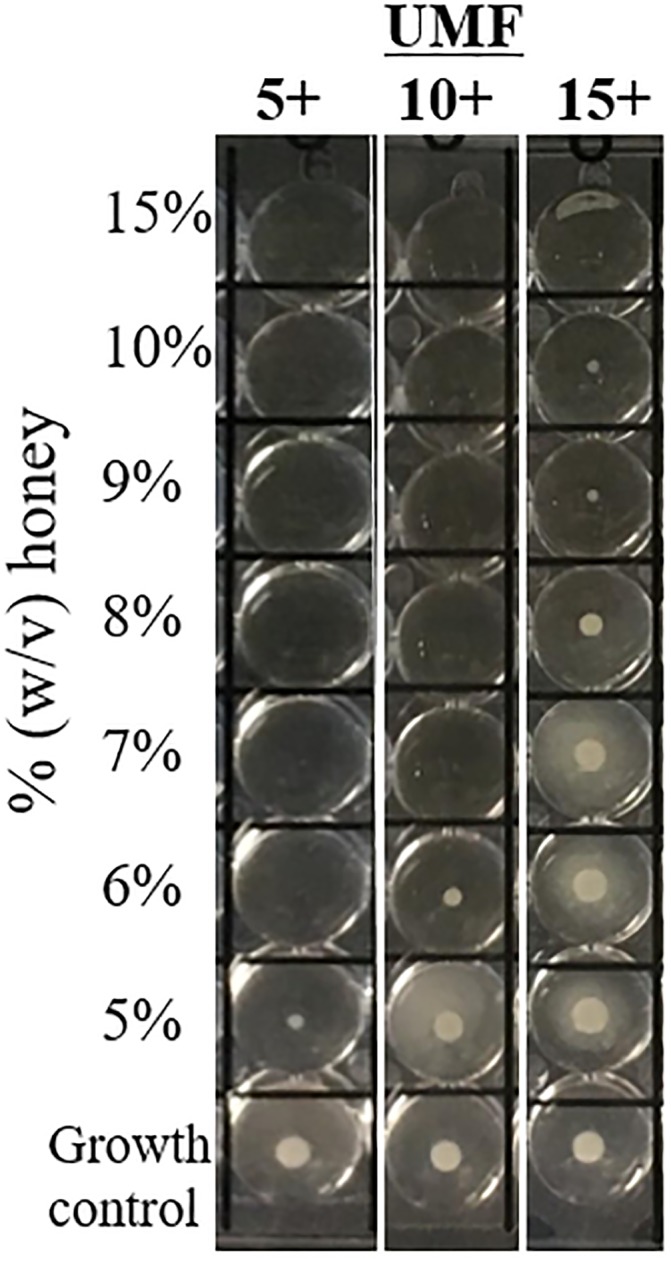
Representative broth microdilution results for an MRSA isolate tested against UMF 5+, 10+, and 15+ honeys. Images of the dilution series for each honey are cropped and shown for side-by-side comparison. Here, the MIC was 6% for UMF 5+, 7% for UMF 10+, and 15% for UMF 10+.

Among the 73 *Staphylococcus* spp., MIC values were significantly lower for UMF 5+ than UMF 10+ (p<0.01), UMF 5+ than UMF 15+ (p<0.01), and UMF 10+ than UMF 15+ (p<0.01). Statistical significance remained (p<0.01) on subset analysis of MRSA (n = 25), MSSA (n = 23), and coagulase-negative staphylococci (n = 25) separately, for which MIC values were significantly lower for UMF 5+ than UMF 10+, UMF 5+ than UMF 15+, and UMF 10+ than UMF 15+. The 5 strains of *S*. *lugdunensis* showed similar MIC values to other coagulase-negative staphylococci, with MIC ranges of ≤5–7% for UMF 5+ and UMF 10+ honey and 6–15% for UMF 15+ honey. There were no significant differences between MIC distributions of MRSA versus MSSA organisms. MIC ranges, MIC_50_ and MIC_90_ values for each honey and staphylococcal group are summarized in Table 1, and example broth microdilution results are shown (Fig 1).

For *Pseudomonas aeruginosa* (n = 22), MIC values were significantly lower for UMF 5+ than UMF 10+ (p<0.05), UMF 5+ than UMF 15+ (p<0.01), and UMF 10+ than UMF 15+ (p = 0.01). Among MDR *P*. *aeruginosa* (n = 10), UMF 5+ yielded lower MIC values than either UMF 10+ honey (p<0.05) or UMF 15+ honey (p<0.05), but UMF 10+ and UMF 15+ MIC values showed no significant difference. Among non-MDR *P*. *aeruginosa* (n = 12), MIC values for both UMF 5+ and UMF 10+ were significantly lower than UMF 15+ honey (p<0.05). MDR strains had significantly lower MIC values than non-MDR strains for UMF 5+ (p = 0.01) and UMF 15+ (p = 0.01) but not UMF 10+ (p = 0.58) honey.

For Enterobacteriaceae (n = 33), MIC values were lower for UMF 5+ than for UMF 15+ honey (p<0.01) and for UMF 10+ than UMF 15+ honey (p<0.01), but not for UMF 5+ compared to UMF 10+ (p>0.05). Compared to non-ESBL/non-carbapenem-resistant Enterobacteriaceae (CRE) organisms (n = 19), ESBL and CRE organisms (n = 14) had higher overall MIC values with UMF 5+ and UMF 10+ honeys (p<0.01), but not UMF 15+ (p = 0.81).

## Discussion

The increasing incidence of multi-drug resistant bacterial infections worldwide poses new challenges which have led to a renewed interest in Manuka honey as an alternative antibiotic agent [3, 18]. Its antibacterial mechanisms, with different target sites, are unique from those of conventional antibiotics, thus Manuka honey could potentially be used as an alternative or ancillary agent in MDR bacterial infections. Through multifactorial mechanisms, Manuka honey has been shown to disrupt the metabolic processes and membrane potential of *S*. *aureus* and *E*. *coli* and the extent of cell viability was dependent on honey concentration [19]. Transcriptomic studies have shown *S*. *aureus* to produce unique expression profiles when exposed to Manuka honey as compared to typical antibiotics [20]. There has furthermore been demonstrated *in vitro* synergism between Manuka honey and conventional antibiotics, as measured by inhibition of bacterial growth or biofilm formation [20–22]. As a topical agent, Manuka honey may be used effectively to treat disorders like atopic dermatitis, blepharitis, rhinosinusitis, and skin ulcers [23–26]. Our data corroborate the measurable antimicrobial activity of Manuka honey against a spectrum of clinical isolates from skin and soft tissue sources, including those with multi-drug resistance such as MRSA, ESBL producers, CRE, and MDR *P*. *aeruginosa* [6, 27–30]. Lower MIC values were achieved against *Staphylococcus* species than with gram-negative pathogens, consistent with the overall trends of prior studies [3]. We additionally demonstrated activity of Manuka honey against *S*. *lugdunensis*, a clinically important coagulase-negative *Staphylococcus*, which to our knowledge has not yet been reported.

Contrary to our expectations, Manuka honey of lower UMF grade demonstrated equal to significantly increased antimicrobial activity compared to higher UMF grade honey for all organism groups tested. While unexpected, this phenomenon has occurred in several other studies. One investigation compared Manuka honey of UMF grades between 5 and 20 against *S*. *aureus* and *E*. *coli* organisms incorporated into tissue engineering scaffolds and found that no significant differences in bacterial clearance regardless of the UMF grade [31]. Other authors have also found that UMF grade did not correlate with antibacterial activity against *P*. *aeruguinosa*, although number of isolates tested was limited [32]. We believe that these findings may be explained by the dynamic nature of the chemical composition of Manuka honey. Dihydroxyacetone (DHA) is the precursor molecule of MGO found in *Leptospermum* flower nectar and by itself lacks antimicrobial activity. With maturation of the honey, a portion of DHA will convert to MGO, thus increasing MGO concentration with time. Decreases in DHA and increases in MGO concentrations begin to occur after Manuka honey extraction, with changes continuing up to at least one year of storage [33, 34]. The extent of DHA conversion to MGO is not wholly predictable for a given sample, as side chemical reactions also occur and predictions are complicated by temperature and other variables [34]. Higher DHA:MGO ratios between 5:1 to 9:1 are observed in fresher Manuka honey compared to lower DHA:MGO ratios approximating 2:1 in older honeys [33, 35]. A major Manuka honey testing laboratory found that final packed Manuka honeys of lower UMF grade tended to have higher DHA:MGO ratios whereas higher grade UMF honeys tended to have lower such ratios and higher content of hydroxymethylfurfural and C4 sugars, indicating honey that was older at the time of UMF grading [35]. Therefore, MGO concentrations and antimicrobial activity at the time of consumer use may not be accurately reflected by UMF labelling. While we did not measure MGO or DHA concentrations of the honeys used during our study, we conjecture that age and storage conditions likely influenced MGO concentrations and resulting antimicrobial activity of the honeys tested.

Manuka honey is marketed as beneficial to health and has been publicized for its antibacterial properties. There is therefore legitimate concern that honeys of higher UMF grades are considered by consumers as higher quality and are sold at premium prices, whereas higher UMF graded honey may not necessarily confer an increased health benefit. Future studies could confirm the variability of *in vitro* antimicrobial efficacy between UMF-graded Manuka honeys from different manufacturers and lot numbers, as our study was limited to single bottles of various UMF grades. MGO and DHA concentrations should also be assessed over time in Manuka honey sold for medicinal purposes and correlated with antibiotic activity to better understand changes in antimicrobial efficacy over its shelf life.

While we detected statistically significant differences in the MIC values, the absolute differences in MICs between the three UMF-graded honeys would be considered small by susceptibility testing standards, generally within two-fold dilutions. It is unknown whether or not these differences in MIC would have a significant clinical impact, such as for topical treatment of wound infections. Studies correlating antimicrobial susceptibility testing results with clinical outcomes are lacking, but may be beneficial to developing best practices in using Manuka honey for its antibiotic activity.

## Conclusions

Manuka honey exhibited antimicrobial activity against a spectrum of MDR and non-MDR bacterial organisms isolated from wound sites, with greater potency against staphylococcal organisms compared to gram-negative bacteria. In a limited sampling, we also found Manuka honey to demonstrate significantly greater antimicrobial activity at lower UMF grades when compared to UMF 15+ honey. We conclude that UMF grade, as an indicator of MGO content and honey quality, may be misleading to the consumer as it may not necessarily correlate with antibacterial efficacy of the Manuka honey at the time of purchase or the time of use. Studies investigating *in vivo* outcomes of Manuka honey of different UMF grades while confirming MGO and DHA content are needed to advance our understanding of use of Manuka honey for medicinal purposes. Despite these concerns, natural products such as Manuka honey are promising as alternative agents in combatting MDR bacterial infections.

## Supporting information

S1 Table Minimal inhibitory concentrations of 3 different UMF grades of Manuka honey for 128 bacterial isolates. Categories and sub-categories of organism as discussed in the text are also indicated. MSSA, methicillin-susceptible *Staphylococcus aureus*; ESBL, extended-spectrum beta-lactamase; KPC, *bla*_*KPC*_ carbapenemase producer; CRE, carbapenem-resistant Enterobacteriaceae; MDR, multi-drug resistant.
(XLSX)
